# Tetrandrine alleviates silicosis by inhibiting canonical and non-canonical NLRP3 inflammasome activation in lung macrophages

**DOI:** 10.1038/s41401-021-00693-6

**Published:** 2021-08-20

**Authors:** Mei-yue Song, Jia-xin Wang, You-liang Sun, Zhi-fa Han, Yi-tian Zhou, Ying Liu, Tian-hui Fan, Zhao-guo Li, Xian-mei Qi, Ya Luo, Pei-ran Yang, Bai-cun Li, Xin-ri Zhang, Jing Wang, Chen Wang

**Affiliations:** 1 Beijing University of Chinese Medicine, Beijing, 100029 China; 2 Department of Pulmonary and Critical Care Medicine, Center of Respiratory Medicine, China-Japan Friendship Hospital, Beijing, 100029 China; 3 National Clinical Research Center for Respiratory Diseases, Beijing, 100029 China; 4 Tsinghua-Peking Center for Life Sciences, Department of Biology, College of Medicine, Tsinghua University, Beijing, 100084 China; 5 School of Pharmaceutical Sciences, Tsinghua University, Beijing, 100084 China; 6 Department of Basic Medical Sciences, School of Medicine, Tsinghua University, Beijing, 100084 China; 7 Institute of Basic Medical Sciences Chinese Academy of Medical Sciences, School of Basic Medicine Peking Union Medical College, Beijing, 100730 China; 8 Peking Union Medical College, Chinese Academy of Medical Sciences, Beijing, 100730 China; 9 Peking Union Medical College, MD Program, Beijing, 100730 China; 10 Department of Respiratory, The Second Affiliated Hospital of Harbin Medical University, Harbin, 150086 China; 11 Department of Pulmonary and Critical Care Medicine, The First Hospital of Shanxi Medical University, Taiyuan, 030001 China

**Keywords:** tetrandrine, silicosis, macrophages, NLRP3 inflammasome, lung function

## Abstract

Silicosis caused by inhalation of silica particles leads to more than ten thousand new occupational exposure-related deaths yearly. Exacerbating this issue, there are currently few drugs reported to effectively treat silicosis. Tetrandrine is the only drug approved for silicosis treatment in China, and despite more than decades of use, its efficacy and mechanisms of action remain largely unknown. Here, in this study, we established silicosis mouse models to investigate the effectiveness of tetrandrine of early and late therapeutic administration. To this end, we used multiple cardiopulmonary function test, as well as markers for inflammation and fibrosis. Moreover, using single cell RNA sequencing and transcriptomics of lung tissue and quantitative microarray analysis of serum from silicosis and control mice, our results provide a novel description of the target pathways for tetrandrine. Specifically, we found that tetrandrine attenuated silicosis by inhibiting both the canonical and non-canonical NLRP3 inflammasome pathways in lung macrophages. Taken together, our work showed that tetrandrine yielded promising results against silicosis-associated inflammation and fibrosis and further lied the groundwork for understanding its molecular targets. Our results also facilitated the wider adoption and development of tetrandirne, potentially accelerating a globally accepted therapeutic strategy for silicosis.

## Introduction

Silicosis is an interstitial lung disease caused by the inhalation of crystalline silica particles and characterized by acute and chronic inflammation with subsequent diffuse fibrosis leading to progressive respiratory insufficiency [1]. It is currently one of the most dangerous occupational diseases worldwide, and responsible for an estimated 10,000 or more new deaths every year [2]. Although protective measures such as dust control and respirators have been applied in attempts to reduce silicosis-associated morbidity, new outbreaks still occur where mining and industrial use of silica materials are prevalent [2]. Unfortunately, aside from lung transplantation, few advances have been made to date in the development of viable treatment options [3]. In China, tetrandrine (Tet) has been approved to treat silicosis although the mechanism of its activity is still largely unclear. Therefore, study of the mechanisms of Tet against silicosis is highly relevant to increasing its effective application and further development of other anti-silicosis candidate drugs.

Tet is a bisbenzylisoquinoline alkaloid extracted from root of *S. tetrandra*, and has been extensively referenced in the Chinese Pharmacopoeia for its use as an anti-silicosis agent [4]. Accumulating in vivo and in vitro evidences suggest that Tet is effective for silicosis treatment [5–7]. Specifically, Tet was reported to minimize the size of well-established silicotic nodules under chest radiograph in a clinical trial including more than 300 patients [8]. However, this potentially exciting effect was not met with uniform acceptance due to insufficient assessment indicators and sample size [8]. In addition to the lack of a standard assessment regimen, the unresolved mechanism of Tet activity has also largely impeded its wider clinical adoption, and its therapeutic efficacy remains controversial. Tet possesses diverse bioactive properties including anti-inflammatory, anti-fibrogenetic, and immunomodulatory effects [4], among others, making it difficult to determine which pathway or molecule that Tet targets to reverse silica-driven inflammation and fibrosis. Systematic and rigorous elucidation of the mechanisms and efficacy of Tet in silicosis is therefore necessary for wider acceptance and clinical application, globally.

The NLRP3 (NLR family pyrin domain-containing 3) inflammasome has been established as a major proinflammatory receptor for sensing environmental danger, such as crystalline silica, and subsequently initiating proinflammatory events during the progression of silicosis [9, 10]. Upon NLRP3 inflammasome activation by crystalline silica, macrophages secrete increasing levels of the proinflammatory factor interleukin (IL)-1β, downstream of the NLRP3 inflammasome. Notably, research in silica-induced animal models has firmly demonstrated that IL-1β secretion exacerbates silicosis, whereas its neutralization can reverse this disease [11, 12]. More importantly, a case report documented that IL-1 receptor blockade dampened the pulmonary inflammation and impeded the progressive deteriorations of respiratory function in silicosis patients [13]. Moreover, multiple studies have reported that Tet has the strong potential to inhibit the release of inflammatory cytokines [5, 14], especially IL-1β [7], which greatly contributes to the pathogenesis of silicosis. Given the sound inhibitory effects of Tet against IL-1β, it is reasonable to speculate that the effectiveness of Tet treatment against silicosis is likely mediated by suppressing activation of the NLRP3 inflammasome. However, the precise genetic or molecular basis by which Tet targets NLRP3 inflammasome signaling pathways requires thorough investigation.

In this work, we employed multiple experimental approaches to assess the early and late therapeutic effectiveness of Tet in silicosis mouse models. In particular, we examined the effects of these treatment regimens on cardiopulmonary function, inflammation, and fibrosis, and confirmed that Tet exerted credible, therapeutic effects on silicosis. In addition, we combined single cell RNAseq and transcriptomics strategies of murine lung tissue to identify the canonical and non-canonical NLPR3 inflammasome signal cascades in pulmonary macrophages as downstream targets of Tet to suppress silicosis-associated inflammation and fibrosis, both in vivo and in vitro. Our study provides a much necessary theoretical framework for understanding how Tet acts in suppression of silicosis and suggests combined therapeutic approaches for improving Tet effectiveness in the clinic.

## Materials and methods

### Reagents

Crystalline silica particles with mass median diameter less than 1.6 µm were obtained from Forsman Scientific (Beijing) Co., Ltd (CAS7631-86-9; purity 99%). These particles were endotoxin-free by baking at 180 °C for at least 2 h and then suspended in the sterile phosphate buffer saline (PBS). All silica suspension was sonicated for at least 15 min and vortexed for 1 min prior to use. Tet with purity higher that 98% was purchased from the Dibo chemical Co., Ltd (Hubei, China) and was dissolved in 1% carboxymethyl cellulose sodium (CMC-Na) to be administered to mice. For cells, stock solutions of Tet were prepared via dissolving Tet in 1 N HCl solution and then adjusting pH to 6.5 with 1 N NaOH. The suspension of Tet was then diluted in Dulbecco’s Modified Eagle’s Medium (DMEM). Suspensions of Tet were sonicated for 20 min and vortexed for 30 s before use.

### Animals

Male C57BL/6 J mice of 8 weeks old were housed in independently ventilated cages under specific pathogen-free conditions and provided fresh water and food per week. They were maintained under a 12/12-h light/dark cycle, with controlled room temperature (23 °C ± 2 °C) and humidity (50%-70%). All the experimental procedures were approved by the Animal Care and Use Committee of Peking Union Medical College.

### Cell culture

Murine peritoneal macrophages were extracted from peritoneal lavage fluid of C57BL/6J mice following intraperitoneal injection of 3% thioglycolate for 72 h. The peritoneal macrophages were cultured in DMEM supplemented with 10% fetal bovine serum (FBS) and 1% penicillin and streptomycin at 37 °C under 5% CO_2_.

### Silicosis model and Tet administration

The silicosis model was established by a single intratracheal exposure of silica suspension as previously delineation [15]. Briefly, mice were anesthetized with 2% pentobarbital (i.p., 0.2 mL/100 g, Sigma-Aldrich) and then 20 μL silica suspension (600 mg of silica in 1 mL of sterile PBS) was delivered into trachea for each mouse. Mice in the sham groups were treated intratracheally with an equal volume of PBS.

To establish an early therapeutic model of Tet administration, mice were randomly divided into five groups (5 mice per group): PBS control group, in which the mice were intratracheally instilled with PBS and gavage administered vehicle (1% CMC-Na) only; PBS + Tet group, in which the mice were intratracheally instilled with PBS and gavage administered Tet (100 mg/kg); silica control group, in which the mice were instilled with silica and gavage administered vehicle (1% CMC-Na) only; silica + LT/HT group, in which the mice were intratracheally instilled with silica and gavage administered low dose (50 mg/kg) or high dose (100 mg/kg) of Tet. These mice in treatment groups were exposed to silica and simultaneously treated with Tet for 4 weeks. For late therapeutic experiments, mice were divided into four groups (9 mice per group): PBS, PBS + Tet, silica, and silica + Tet (100 mg/kg) groups, in which the mice were treated same as their counterparts in the early therapeutic model. Unsimilarly, these mice were administered with the indicated agents once a day for 4 weeks beginning at day 14 after silica exposure. Subsequent mechanism experiments were also conducted in this late therapeutic silicosis model.

To investigate inhibition of NLRP3 inflammasome by Tet exerted in macrophages, peritoneal macrophages were divided into four groups: PBS, PBS + Tet (100 μM), silica (50 µg/cm^2^) and silica (50 µg/cm^2^) + Tet (100 μM) groups. These macrophages were treated with indicated agents and incubated for 3 h. Cells and cell supernatant were collected and then assayed.

### Lung function test

Lung function test was performed using FlexiVent apparatus with the forced oscillation system (SCIREQ, Montreal, Quebec, Canada) following the manufacturer’s protocol. Briefly, after tracheotomy, tracheas of all deeply anesthetized mice were connected with an auto-ventilator by an endotracheal tube. Then, resistance (Rrs), compliance (Crs), and elastance (Ers) of the whole respiratory system (lung, airways and chest wall) were obtained under “snapshot perturbation” maneuver, tissue damping (resistance) G and H under forced oscillation perturbation (primewave-8) maneuver, static compliance (Cst) and inspiratory capacity (IC) under maximal PV loops maneuver. All maneuvers and perturbations were conducted until numerical stability and two values were achieved.

### Hemodynamic measurements

Right ventricular systolic pressure (RVSP) was evaluated according to the modified Bernoulli equation and was identical to the pulmonary artery systolic pressure (PASP) when the right ventricular outflow obstruction was absent [16]. Invasive RVSP was directly measured by inserting the right ventricle with an eight-gauge needle that was connected to the high-fidelity pressure transducer. The RVSP was recorded and analyzed by Power Lab Data Acquisition and Analysis System (PL 3504, AD Instruments, Australia). The lung and heart were then removed and separated. Right ventricular hypertrophy index (RVHI) was quantified by the Fulton index measurement that the ratio is calculated by right ventricle over the sum of left ventricle plus septum (RV/LV + S).

### Bronchoalveolar lavage fluid collection

Bronchoalveolar lavage fluid (BALF) was obtained as previously reported [15]. In brief, after the right bronchus was clamped, the left lung lobe was lavaged with 0.4 mL of 0.9% NaCl for three times to recover the BALF. The samples were subsequently placed on ice and were processed within 2 h. Samples were centrifuged at 800 r/min for 5 min at 4 °C and the supernatant was stored at −80 °C until analysis. The deposited cells were washed and re-suspended with 300 μL of cold PBS for differential cell population count by Grunewald-Giemsa staining and total cell count by automatic cell counter.

### Histological analysis

The left lung samples were fixed into 4% paraformaldehyde and then embedded in paraffin. Sample blocks were sectioned (5 μM per section) for Hematoxylin and eosin (HE) staining to evaluate inflammation by Szapiel’s method [17], Masson’s trichrome staining to assess the fibrotic degree according to King’s method [18]. All sections after stanning were visualized using 3D HISTECH digital slice scanner (Hungary). The assessment of fibrotic lesions was determined by Fibrotic Lesion Scores that applied as previously described [15]. In detail, distinct silicotic nodules were classified according to King’s method and different weight ranging 0-5 was determined. Then Fibrotic Lesion Score was calculated as the weight levels (0-5) multiplied by their corresponding percentage of fibrotic area over the total area of the tissue section. For immunohistochemistry (IHC), murine lung sections were stained with the anti-collagen-I antibody (1/200 dilution) at 4 °C overnight, then washed and incubated with secondary antibody for 1 h at room temperature. Sirius Red staining was performed by incubating slides in 0.1% Sirius Red F3B for 1 h, washing twice in acidified water, dehydrating thrice in 100% ethanol, and then clearing in xylene. The percentage of positive stained areas by IHC and Sirus Red staining was blindly determined by two pathologists and reported as percentage.

### Hydroxyproline assay

The measurement of hydroxyproline was detected with a hydroxyproline (HYP) measurement kit (NBP2-59747, Novus Biologicals, Littleton, CO, USA) rigorously based on the manufacturer’s instructions. Approximately 20 mg (wet weight) lung tissue of each mouse was collected to detect. The absorbance of 560 nm was measured and the hydroxyproline content was determined against standards of HYP.

### Quantitative PCR

According to the manufacturer’s instructions, total RNA from lung tissue or cell samples was extracted using TRIzol reagent (Invitrogen, Carlsbad, CA, USA). Complementary DNA (cDNA) was synthesized by reverse transcription with a Tiangen kit (KR103, Tiangen Biotechnology, Beijing, China). Gene expression was analyzed by quantitative PCR assay. The PCR amplification was performed in triplicate using SYBR Green I Q-PCR kit (TransGen Biotech, Beijing, China). Data acquisition and analysis of quantitative PCR assay were conducted in a Bio-Rad IQ5 system (Bio-Rad, Hercules, CA, USA). To normalize non-PCR-related fluorescence fluctuations between wells, each fluorescent reporter signal was detected against the internal reference dye signal of β-Actin. Sequences of primers used in our experiment were listed in Supplementary Table1. All primers were synthesized by Beijing Tianyihuiyuan Biotechnology Company.

### Western blot

The total protein was extracted with RIPA lysis buffer from lung tissue (P0013B, Beyotime, Shanghai, China). Protein concentration of samples was detected using BCA Protein Assay Kit (23225, Thermo Fisher Scientific, Waltham, MA, USA). Protein samples were separated on an 8%-15% SDS-polyacrylamide gel electrophoresis gel and transferred onto polyvinylidene difluoride (PVDF) membranes. Tanon Automatic Chemiluminescence/Fluorescence Image Analysis System (5200, Tanon, Shanghai, China) was used for protein signal detection. Protein relative expression level was analyzed by ImageJ software and calculated as: band gray value/internal parameter (Actin) gray value. Antibodies are described in the supplementary materials.

### ELISA and Quantitative inflammatory microarray

The protein levels of IL-1β and IL-18 in BALF and serum, as well as IL-6 in BALF were detected by ELISA kits according to the manufacturer’s instructions. Specific kits information was listed in the supplementary materials.

The profiles of 308 cytokines in serum of mice in blank PBS group, silica group and silica + Tet group were simultaneously detected using a mouse L308 quantitative microarray (AAM-BLG-1-4, RayBiotech, Guangzhou, China) according to the manufacturer’s instructions. The cytokines with significantly distinct expression were screened out and analyzed.

### RNA sequencing

RNA sequencing (RNA-seq) was conducted using lung tissues from PBS mice (*n* = 3), silicosis mice (*n* = 3) and Tet-administered silicosis mice in late therapeutic administration model (*n* = 3). Paired-end reads of 150 bp length were generated using Illumina Hiseq platform. Cleaned reads were obtained by excluding adaptors and lower quality reads, and were then mapped to Ensembl GRCm38 mouse reference genome by Hisat2 (v.2.1.0) with specifying option “–dta”. Samtools (v.1.9) was used to sort mapped reads and transform decimal sam files to binary bam files. Sorted reads were assigned to Ensembl GRCm38.90 mouse annotated genomes by Stringtie (v.2.0) with specifying parameter “-e”. Gene expression of matrix was analyzed by python script prepDE.py. Normalization and gene differential expression were analyzed by R package DEseq2 (v.3.10). The differentially expressed genes were obtained within PBS group versus silicosis group and silicosis group versus Tet-administered group, respectively.

### Single cell RNA sequencing

Four samples from PBS control and three samples from silica models (treated with silica for 6 weeks) were used for single cell RNA-seq using 10×Genomics platform. Cell Ranger (v3.0.2) software from 10X genomics and scrublet Python package were hired to perform read alignment and quality control. The samples were integrated and clustered using Seurat R package (v3.1.4). Approximately 51,000 single cells passed QC and annotated to 18 distinct clusters based on gene expression patterns of each cell.

### Statistical analysis

All statistics were performed using Prism 8.0 software. For all the graphs, data were represented as mean ± SEM (standard error of the mean), *P* value < 0.05 was considered statistically significant. Before analysis, normality tests were conducted for all data. Comparisons between multiple groups were carried out using two-ways ANOVA followed by Tukey *post hoc*-test. Nonparametric tests were applied to data that were not normally distributed.

## Results

### Tet treatment improved lung impairment, inflammation, and fibrosis in an early therapeutic silicosis mouse model

**Fig. 1:**
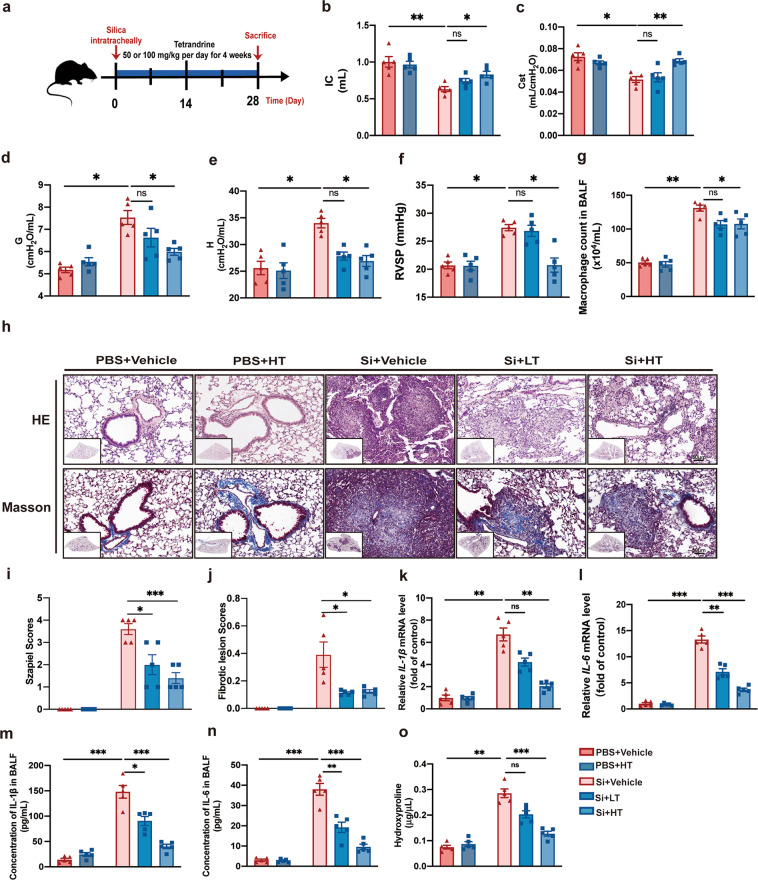
Tet treatment improved lung impairment, pulmonary inflammation, and fibrosis in an early therapeutic silicosis mouse model. **a** Schematic of Tet administration in an early therapeutic silicosis mouse model. There are five groups (PBS + Vehicle, PBS + HT, Si + Vehicle, Si + LT, and Si + HT groups) in the following experiments of Fig. 1 (5 mice per group). LT, low dose of Tet (50 mg/kg); HT, high dose of Tet (100 mg/kg). **b-e** Lung function test from mice treated as in (**a**). IC, inspiratory capacity; Cst, static compliance; G, H, tissue damping (resistance). **f** Right ventricular systolic pressure (RVSP) from mice treated as in (**a**). **g** Macrophage count in bronchoalveolar lavage fluid (BALF) from mice. **h** Representative images of hematoxylin and eosin (HE) staining (above) and Masson staining (below) of lung sections from mice treated as in (**a**) (*n* = 5 per group). Scale bar, 50 μm. **i** Statistical analysis of Szapiel Scores of HE staining in images of (**h**). **j** Statistical analysis of Fibrotic lesion Scores of Masson staining in images of (**h**). **k** mRNA level of *IL-1β* in lung tissue from mice. **l** mRNA level of *IL-6* in lung tissue from mice. **m** Concentration of IL-1β in BALF from mice. **n** Concentration of IL-6 in BALF from mice. **o** Hydroxyproline assay of lung tissue from mice. The data are reported as the mean ± SEM. Significance for each figure: *P < 0.05, **P < 0.01, ***P < 0.001 and ns indicates not significant.

In order to evaluate the effects of Tet administration systematically and comprehensively prior to silicotic fibrosis we examined lung function, inflammatory response, and fibrosis development in a silicosis mouse model treated with Tet. To establish the silicosis mouse model, we applied a single intratracheal instillation of silica suspension in 8-week-old male C57BL/6 J mice. At the time of silica instillation, mice were also given intragastric Tet for 4 weeks until sacrifice (Fig. 1a). Mice were evaluated prior to sacrifice. We found that, compared with untreated controls, high-dose Tet administration, which is superior to low-dose Tet, resulted in attenuation of silica-induced impairment of lung function, based on several indicators including inspiratory capacity (IC) (Fig. 1b), static compliance of the respirable system (Cst) (Fig. 1c), tissue damping (resistance) G (Fig. 1d), H (Fig. 1e), compliance (Crs) (Fig. S1a). However, both low- and high-dose Tet both  reduced elastance (Ers) (Fig. S1b), and only low-dose Tet exhibited good outcome on resistance (Rrs) (Fig. S1c) of the respirable system. Previous studies showed that silicosis could dramatically increase the risk of pulmonary hypertension in patients and mice [19–21]. Thus, we evaluated right ventricular systolic pressure (RVSP) (Fig. 1f) and right ventricular hypertrophy index (RVHI) (Fig. S1d) and found RVSP was reduced in high dose of Tet-treated silicosis mice, whereas RVHI exhibited a decreasing trend with no statistical significance.

Immune cells, especially macrophages associated with lung inflammation were also remarkably increased in untreated silicosis mice compared with the PBS control group, as indicated by higher macrophages (Fig. 1g), lymphocytes (Fig. S1e), neutrophils (Fig. S1f), and total cell count (Fig. S1h) in bronchoalveolar lavage fluid (BALF). Among them, macrophages count in BALF was reduced by Tet treatment. Furthermore, hematoxylin and eosin (HE) staining (Fig. 1h, i) of lung sections indicated more severe diffuse alveolitis in the silicosis group than in the Tet-treatment group. Likewise, mRNA and protein levels of IL-1β (Fig. 1k, m) and IL-6 (Fig. 1l, n) were both down-regulated under Tet treatment. We next examined the level of pulmonary fibrosis between silicosis and Tet-treated lungs using several tissue staining techniques. Masson’s trichrome staining (Fig. 1h, j) revealed a less degree of collagen deposition in the Tet-treated silicosis lungs than in the silicosis lungs. Similarly, collagen-I staining by both Sirius Red (Fig. S1g, j) and immunohistochemistry (IHC) (Fig. S1g, i) also confirmed the amelioration of fibrotic state in lung tissue from Tet treatment group, when compared to their untreated silicosis counterparts. Hydroxyproline assay also showed that high-dose Tet dramatically reduced the collagen deposited in silicosis mice (Fig. 1o). Taken together, these results showed that Tet treatment resulted in a dose-dependent reduction in pulmonary inflammation and fibrosis symptoms, as well as lung function impairment in silicosis mouse model.

### Late Tet therapeutic treatment reduced inflammation and fibrosis and partially ameliorated impaired lung function in silicosis mice

**Fig. 2:**
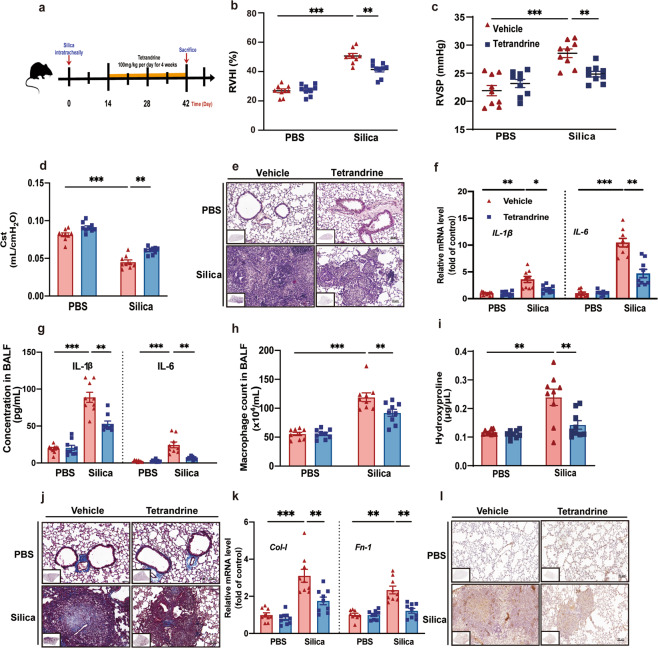
Late Tet therapeutic treatment reduced pulmonary inflammation and fibrosis and partially ameliorated impaired lung function in silicosis mice. **a** Schematic of Tet administration in late therapeutic administration model of silicosis mice. There are four groups (PBS + Vehicle, PBS + Tet, Si + Vehicle, Si + Tet groups) in the following experiments of Fig. 2 (9 mice per group). **b**, **c** RVHI, RVSP of mice treated as in (**a**). **d** Lung function test of Cst of mice. **e** HE staining of murine lung sections from mice treated as in (**a**) (*n* = 9 per group). **f** mRNA levels of *IL-1β* and *IL-6* of lung tissue from mice. **g** Concentration of IL-1β and IL-6 in BALF from mice. **h** Macrophage count in BALF from mice. **i** Hydroxyproline assay of lung tissue from mice. **j** Masson staining of murine lung sections from mice treated as in (**a**) (*n* = 9 per group). **k** mRNA levels of *Col-I* and *Fn-1* in lung tissue from mice. Fn-1, Fibronectin-1. **l** IHC staining of Col-I of lung sections from mice treated as in (**a**) (*n* = 9 per group). The data are reported as the mean ± SEM. Significance for each figure: *P < 0.05, **P < 0.01, ***P < 0.001 and ns indicates not significant.

We next investigated the late therapeutic effects of Tet administration after the formation of silicotic nodules in silicosis mouse by evaluating cardiopulmonary function, inflammation, and fibrosis. Considering the former result, we decided to select the high dose of Tet (100 mg/kg) with better efficacy in this late therapeutic administration model. To this end, we treated C57BL/6 J mice with Tet for 4 weeks starting at day 14 after silica exposure (Fig. 2a). We first measured right ventricular function through RVHI (Fig. 2b), RVSP (Fig. 2c, Fig. S2a), which revealed that late Tet treatment could lead to decreased RVSP and RVHI. In addition, Tet upregulated Cst (Fig. 2d) significantly and improved Crs (Fig. S2e) and IC (Fig. S2b) in an increasing trend with no significance. Tet down-regulated Rrs (Fig. S2f) significantly, whereas decreased G (Fig. S2c) and H (Fig. S2d) in a trend with no significance in Tet-administered silicosis mice. Collectively, these data suggest that Tet has limited improved effects on cardiopulmonary function when administered after onset in silicosis mice.

We also examined levels of pulmonary inflammation in murine lungs treated with Tet at 2 weeks after silicosis induction. HE staining (Fig. 2e) and subsequent quantitative image analysis of alveolitis showed higher Szapiel scores (Fig. S2g) induced by silica and mitigation of this effect in the Tet treatment group. We also assessed the transcriptional expression of IL-1β, IL-6, which are inflammatory cytokines commonly used as markers for inflammatory response. In agreement with Szapiel scores, we found that both mRNA levels (Fig. 2f) and protein concentration (Fig. 2g) of IL-1β and IL-6 were all increased in silicosis mice, but were decreased in that of the Tet-treated silicosis mice. The levels of macrophage (Fig. 2h), neutrophil (Fig. S2h), lymphocyte (Fig. S2i) and total cell (Fig. S2j) count in BALF were also decreased by Tet treatment. In addition, we also determined the anti-fibrotic effects of Tet treatment at 2 weeks after the onset of silicotic nodules using hydroxyproline assays (Fig. 2i), Masson’s trichrome staining (Fig. 2j, S2k), and IHC staining of collagen-I (Fig. 2l, S2l), which all showed a substantial reduction in these markers with Tet administration, compared with untreated silicosis mice. In addition, reduced collagen-I and fibronectin-1 mRNA levels (Fig. 2k) and fibronectin-1 protein levels (Fig. S2m) were consistent with the above results. Cumulatively, these experimental results demonstrated that lung inflammation and fibrosis could be substantially attenuated by Tet administration, while cardiopulmonary function could be partially improved in silicosis mouse lungs.

### Tet suppressed NLRP3 inflammasome activation induced by silica in vivo

**Fig. 3:**
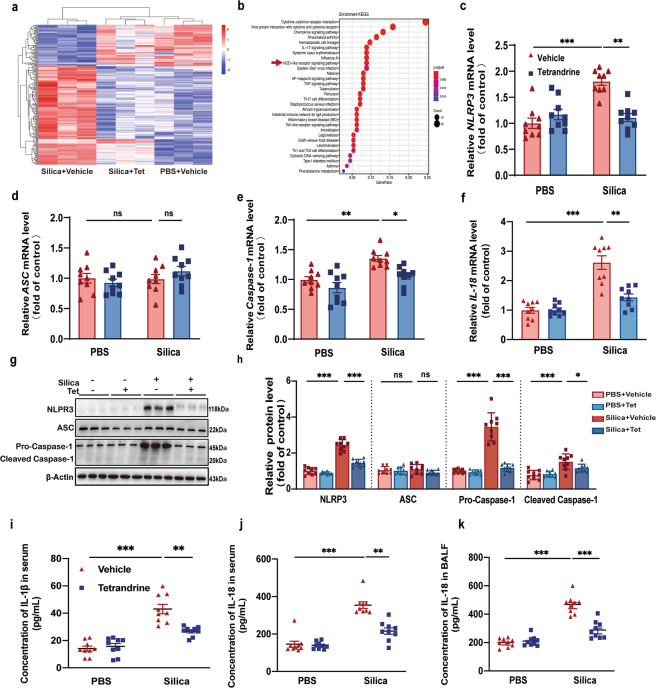
Tet suppressed NLRP3 inflammasome activation induced by silica in vivo. **a** Hierarchical clustering heatmaps of significant differentially expressed genes in Si, Si + Tet, and PBS groups of mice treated as Fig. 2a (*n* = 3 per group). **b** Functional enrichment analysis with KEGG biological processes was performed using KEGG database in Si, Si + Tet, and PBS groups of mice treated as Fig. 2a (*n* = 3 per group). **c-f** mRNA levels of *NLRP3*, *ASC*, *Caspase-1*, and *IL-18* of lung tissue from mice treated as Fig. 2a (*n* = 9 per group). **g** Western blots of NLRP3, ASC, Pro-Caspase-1, and Cleaved Caspase-1 of lung tissue from mice treated as Fig. 2a (*n* = 9 per group). **h** Statistical analysis of band densities from images in (**g**). **i, j** Concentration of IL-1β and IL-18 in serum of mice. **k** Concentration of IL-18 in BALF of mice. The data are reported as the mean ± SEM. Significance for each figure: *P < 0.05, **P < 0.01, ***P < 0.001 and ns indicates not significant.

Despite the apparently ameliorative effects of Tet administration observed in silicosis mice, understanding of its mechanism of action remains limited. Hence, we performed RNA sequencing (RNAseq) followed by hierarchical clustering of samples and estimation of differentially expressed genes (DEGs) among lungs of the Si, Si + Tet and PBS mice groups of late administration models (Fig. 3a). The RNAseq analysis revealed that the cytokine, chemokine, Th17, and NOD-like receptor signaling pathways, among others that were the top 30 aberrent pathways contributing to the development of silicosis, were significantly affected by Tet treatment (Fig. 3b). Among these, NLRP3 inflammasome activation that is part of the NOD-like receptor signaling pathway has been reported to participate in the pathogenesis of silicosis [22]. We therefore hypothesized that the therapeutic anti-silicosis effects of Tet may be meditated by inhibiting the activation of NLRP3 inflammasome. To explore this possibility, we used real-time quantitative PCR (qPCR) to detect the relative expression of several essential genes in the NLRP3 inflammasome pathway including NLRP3, ASC, and Caspase-1 (Fig. 3c-e) and Western blots to determine the levels of protein accumulation (Fig. 3g, h) in lung tissue from late administration mouse models, which indicated that the strong activation of NLRP3 inflammasome was repressed by Tet in silicosis mice, although no significant change in ASC mRNA and protein levels was observed. Moreover, mRNA level of IL-1β (Fig. 2f) and concentration in BALF (Fig. 2g) and serum (Fig. 3i) were down-regulated by Tet administration. Similar results were observed in IL-18 at both mRNA (Fig. 3f) and protein levels (Fig. 3j, k). These results strongly suggested that Tet administration inhibited the activation of NLRP3 inflammasome induced by silica exposure in vivo.

### Tet inhibited NLRP3 inflammasome activation by canonical and non-canonical pathways in vivo

**Fig. 4:**
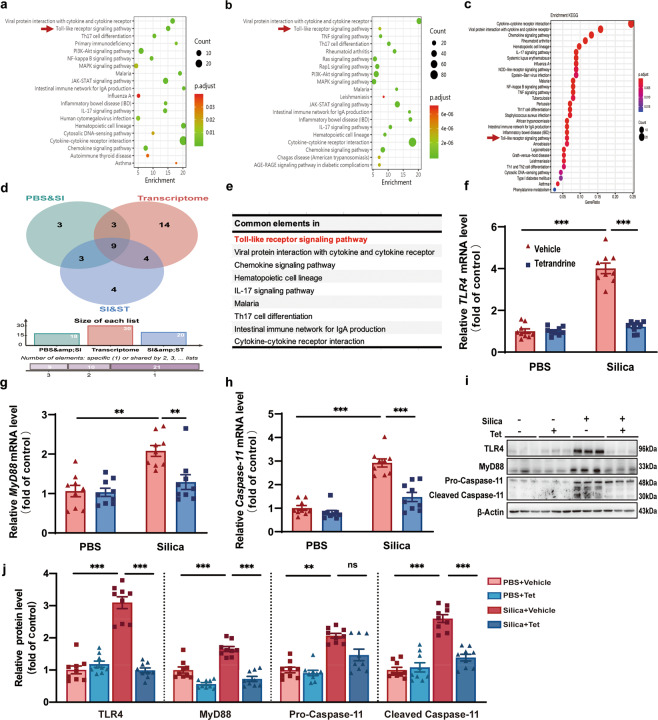
Tet inhibited NLRP3 inflammasome activation by canonical and non-canonical pathways in vivo. **a** Functional enrichment analysis with KEGG biological processes of the significant different inflammatory protein in serum identified by quantitative inflammation microarray from control mice and silicosis mice. The top 20 pathways with a *P* < 0.05 were shown with their names. **b** Functional enrichment analysis with KEGG biological processes of the significantly different inflammatory protein in serum identified by quantitative inflammation microarray from silicosis mice treated with or without Tet. The top 20 pathways with a *P* < 0.05 were shown with their names. **c** Functional enrichment analysis with KEGG biological processes was performed using KEGG database in Si, Si + Tet, and PBS groups of mice treated as Fig. 2a (*n* = 3 per group). **d** The Venn diagram of transcriptome and quantitative inflammation microarray analysis. The intersecting signaling pathways were listed in the table in Fig. 4**e**. **f-h** The mRNA levels of *TLR4, MyD88*, and *Caspase-11* of murine lung tissue in four groups (PBS + Vehicle, PBS + Tet, Si + Vehicle, and Si + Tet groups) treated as Fig. 2a (*n* = 9 per group). **i** Western blots of TLR4, MyD88, Pro-Caspase-11 and Cleaved Caspase-11 of murine lung tissue treated as (**f**). **j** Statistical analysis of band densities from images in (**i**). The data are reported as the mean ± SEM. Significance for each figure: **P < 0.01, ***P < 0.001 and ns indicates not significant.

NLRP3 inflammasome activation is mediated by both canonical and non-canonical signaling pathways. The canonical pathway is co-stimulated by priming and activating signal cascades. The priming signal triggers the TLR4 (Toll-like receptor 4) signaling pathway to initiate the production of precursors such as IL-1β and IL-18; the activating signal prompts the assembly of the NLRP3/ASC/Pro-Caspase-1 protein complex [23]. By contrast, non-canonical NLRP3 inflammasome activation is mediated by Caspase-11 in mice and Caspase-4 and Caspase-5 in human [24]. In our study, we conducted quantitative inflammatory microarray analysis in serum between PBS and Silica groups (Fig. 4a) as well as between Silica and Silica + Tet groups (Fig. 4b), combined with whole transcriptome RNAseq in PBS, Silica, and Silica + Tet groups (Fig. 4c) to identify which pathways mediate NLRP3 inflammasome activation in silicosis mice. Intersection in Venn diagram is shown for the enriched pathways by multi-omics analyses (Fig. 4d), containing Toll-like receptor (TLR) signaling pathway, chemokine, cytokine-cytokine receptor interaction and so on (Fig. 4e). Previous reports that the pro-forms of NLRP3 inflammasome associated cytokines were typically generated through downstream transcriptional regulation of TLR expressions, combined with Venn diagram analysis, suggested that Tet interfered with the Toll-like receptor signaling pathway to inhibit NLRP3 inflammasome activation. We verified this possibility by detecting mRNA (Fig. 4f, g) and protein (Fig. 4i, j) levels of several essential gene factors in the TLR4-MyD88 pathway and found that these markers in silicosis mice declined under Tet treatment. Surprisingly, in addition to blocking the priming step, we observed that Tet treatment could also suppress the canonical activating process mediated by Caspase-1 (Fig. 3e, g, h). Furthermore, we found that Tet reduced mRNA and protein levels of Caspase-11 (Fig. 4h-j), indicating that it also blocked the non-canonical NLRP3 inflammasome activation pathway. Collectively, these results showed that both the canonical and non-canonical pathways of NLRP3 inflammasome in silicosis were blocked by Tet administration, indicating that its effects in ameliorating silicosis were likely due to suppressing upstream of canonical and non-canonical NLRP3 inflammasome signaling.

### Tet restrained NLRP3 inflammasome activation in macrophages

**Fig. 5:**
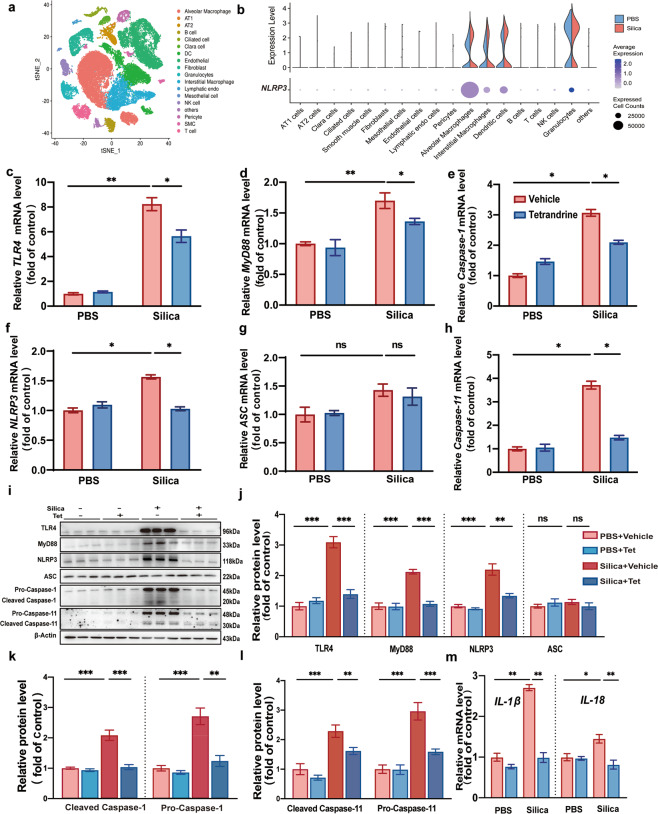
Tet restrained NLRP3 inflammasome activation in macrophages. **a** Cell cluster analysis in PBS and silicosis murine lung tissue. **b** Violin plots representing heterogeneity in expression of NLRP3 in different cell types from control and silicosis lung tissue. **c-h** The mRNA levels of *TLR4*, *MyD88*, *Caspase-1*, *NLRP3*, *ASC*, and *Caspase-11* in macrophages from four groups (PBS + Vehicle, PBS + Tet, Si + Vehicle, and Si + Tet groups). **i** Western blots of TLR4, MyD88, NLRP3, ASC, Pro-Caspase-1, Cleaved Caspase-1, Pro-Caspase-11, and Cleaved Caspase-11 in macrophages in four groups. **j–l** Statistical analysis of band densities from images in (**i**). **m** mRNA levels of *IL-1β* and *IL-18* in macrophages in four groups. The data are reported as the mean ± SEM. Significance for each figure: *P < 0.05, **P < 0.01, ***P < 0.001 and ns indicates not significant.

According to single cell RNAseq analysis from control and silicosis murine lung tissue, it revealed that NLRP3 expression is mainly restricted to macrophages among 18 different cell populations (Fig. 5a, b). Hence, we proposed that suppression of NLRP3 inflammasomes contributed to the anti-silicosis effects of Tet that mainly occurred in macrophages. We first examined the mRNA (Fig. 5c, d) and protein (Fig. 5i, j) levels of canonical pathway priming genes and found that they were downregulated under Tet treatment in primary macrophages exposed to silica, suggesting that Tet inhibited the regulatory effects of TLR4 and MyD88 in these cells. In addition, Caspase-1 and NLRP3 mRNA (Fig. 5e, f) and protein (Fig. 5i–k) levels in the canonical activating step were consistent with the transcriptional down-regulation observed in the priming step. However, surprisingly, we found that ASC mRNA (Fig. 5g) and protein (Fig. 5i, j) levels were unaltered in both Tet-treated and untreated silicosis mice compared to non-silicosis controls. Non-canonical pathway mediated by Caspase-11 was also determined by qPCR (Fig. 5h) and Western blots (Fig. 5i, l) and the results showed that Caspase-11 is down-regulated by Tet treatment in silicosis macrophages. Moreover, the characteristic up-regulation in IL-1β and IL-18 (Fig. 5m) inflammatory cytokines associated with silicosis was also substantially mitigated in macrophages under Tet treatment. In summary, we confirmed a novel mechanism by which Tet acts in macrophages to suppress NLRP3 inflammasome activation via canonical and non-canonical signaling pathways both in vitro and in vivo, which results in attenuation of pulmonary inflammation and subsequent fibrosis.

## Discussion

In this study, we used multiple methods to investigate the efficacy of Tet in both early and late therapeutic administration models of silicosis mice, which resulted in functional, pathological, and molecular level improvements of lung. Using multi-omics techniques, further investigation provided the first evidence that pharmacological repression of NLRP3 inflammasome activation by Tet was likely responsible for the positive treatment outcomes. Taken together, our work lends strong evidence supporting the benefits of Tet in limiting inflammatory conditions accompanying silicosis and provides valuable insights into the mechanisms underlying this disease attenuation.

Tet, a plant-based extract of *Stephania tetrandra* S. Moore, has already been approved for and applied in clinical treatment of silicosis [4, 8]. In fact, to date, albeit with unclear mechanisms underlying the attenuation of silicosis, this drug remains the only pharmacological treatment for silicosis approved by the National Medical Products Administration. Intriguingly, several clinical trials have reported that patients with Tet treatment have exhibited smaller silicotic nodules under X-ray, and have down-regulated inflammatory cytokines and improved lung function compared to their non-Tet treatment counterparts [6]. Our work supports these findings and further confirms that both early and late application of Tet shows favorable outcomes indicating that Tet could dampen silicosis-related inflammation and halt diffuse fibrosis in an experimental mouse model.

Although Tet has previously been reported to be effective in both experimental models and silicotic patients and has a decades-long record of clinical application in China, it has still not been systematically and rigorously investigated as an effective agent for silicosis treatment. In fact, the mechanism by which Tet attenuates silicosis has eluded researchers for several decades, although generally the inhibition of pathological collagen-associated gene expression and inactivation of inflammatory effector cells have been attributed to the Tet-mediated attenuation of silicosis in animal models [6, 25, 26]. However, the specific aberrant factors or pathways that Tet targets remain unclear. NLRP3 inflammasomes in macrophages are essential for silica recognition and initiated subsequent profibrotic events that have been implicated in the pathogenesis of silicosis. Alveolar macrophages are in continuous interaction with the inhaled silica particulates during their clearance, which results in robust release of IL-1β, in an NLRP3 inflammasome-dependent manner [10, 22]. Substantial evidence supports that macrophages stimulated with silica exhibit high levels of IL-1β and that silica-induced fibrosis does not develop in IL-1R-deficient silicosis mice [27, 28]. Likewise, mice with ASC or NLRP3 knockout are more resistant to developing silica-induced chronic fibrosis. In addition, macrophages lacking these NLRP3 inflammasome components are incapable of releasing proinflammatory IL-1β [22]. In light of previous findings that Tet showed high affinity binding to alveolar macrophages and strongly inhibited production of IL-1β in macrophages [29, 30], we thus speculated that the attenuation of silicosis by Tet might be ascribed to upstream blockade or inhibition of IL-1β and/or NLRP3 inflammasome.

In this study, our work showed that Tet could inhibit both canonical and non-canonical NLRP3 inflammasome pathways, suggesting that Tet likely targets a shared upstream component (or components) (Fig. 5). However, the specific aberrant protein or factor that Tet targets remains unclear. Recently, Peeters et al. reported that particle uptake was pivotal to silica-induced inflammasome activation [30]. Also, they showed that cytochalasin D, an inhibitor that specifically blocks actin polymerization, dramatically attenuated the silica-induced secretion of cytokines by NLRP3 inflammasome activation. These findings indicated that cytoskeleton-mediated silica internalization is indispensable for NLRP3 inflammasome activation. Most strikingly, several studies consistently determined that Tet exhibited cytoskeletal depolymerization activity. For example, Chen et al. reported that Tet moderately interrupted the process of collagen biosynthesis by hindering the movement of cytoskeleton to eliminate intracellular procollagen [31, 32]. Lv et al. also found that Tet inhibited the migration and invasion of rheumatoid arthritis fibroblast-like synoviocytes through down-regulation of the key signaling nexus of cytoskeleton formation [33]. In light of these previous studies, we hypothesized that Tet may serve as an initial blocker to prevent silica particle internalization, thus resulting in extensive inhibition of several NLRP3 inflammasome pathway components. In addition, a very recent study also determined that Tet attenuated cerebral ischemia/reperfusion injury by inhibiting NLRP3 via Sirt-1, which silenced many key genes involved in this NLRP3 pathway [34]. These findings indicated that even if the silica particles were internalized by macrophages, Tet could still prevent the activation of NLRP3 by silencing key genes. Further investigations of the specific mechanisms by which Tet regulates the cytoskeleton and Sirt-1 are necessary to definitively understand its targets in silicosis.

In this study, we confirmed the anti-inflammatory and anti-fibrotic effects of Tet based on multiple lines of evidence, including single cell and whole lung RNAseq, quantitative inflammatory microarray of serum and multiple treatments in murine models of silicosis. However, further rigorous and statistically sound confirmation of the clinical benefits of Tet in silicosis patients requires large-scale, longitudinal cohort clinical trials. In addition, mice deficient in components of the NLPR3 pathways are also essential to identify with certainty whether the effects of Tet treatment are dependent on the inactivation of NLRP3 inflammasomes.

## Conclusion

Using early and late administration strategies, our work demonstrated that Tet can effectively attenuate silica-induced pulmonary inflammation, fibrosis, and impairment of lung function in mice. In addition, based on multiple transcriptomics approaches, we provide the first report of Tet-mediated alleviation of silicosis via inhibition of NLRP3 inflammasome pathway signaling. The results of our study provide a substantial conceptual framework for understanding the basic mechanisms by which Tet ameliorates silicosis. Furthermore, our work also provides strong evidence for clinical strategies that combine Tet with NLRP3 inflammasome pathway inhibitors to increase the efficacy and therapeutic range available for silicosis patients.

## Supplementary information

Supplementary material

Supplementary Figure S1

Supplementary Figure S2

Supplementary Figure legend
