# Supplementary material for: Tetrandrine alleviates silicosis by inhibiting canonical and non-canonical NLRP3 inflammasome activation in lung macrophages
Source: Acta Pharmacol Sin. 2021 Aug 20;43(5):1274–84. doi: 10.1038/s41401-021-00693-6 (PMC9061833; doi:10.1038/s41401-021-00693-6)
Supplement: Supplementary file 1 — Supplementary material [file 41401_2021_693_MOESM1_ESM.docx]

| **Supplementary material Table 1** | |  |
| --- | --- | --- |
| Primers for Quantitative Real-Time PCR. | |  |
| Gene |  | Sequence 5’----3’(Mouse) |
| β-Actin | Foward | CATTGCTGACAGGATGCAGAAGG |
|  | Reverse | TGCTGGAAGGTGGACAGTGAGG |
| Caspase-1 | Foward | CCGCGGTTGAATCCTTTTCA |
|  | Reverse | AGTTCCTTTCCAACAGGGCG |
| Caspase-11 | Foward | ACTCTGGAGAAATGTGGATCAGA |
|  | Reverse | AGCCTCCTGTTTTGTCTCGG |
| IL-1β | Foward | ACCTAGCTGTCAACGTGTGG |
|  | Reverse | TCAAAGCAATGTGCTGGTGC |
| IL-18 | Foward | AACACTGGCTGTTCCCACAA |
|  | Reverse | CGGGGCCTGAGGATTATAGC |
| IL-6 | Foward | CTCATTCTGCTCTGGAGCCC |
|  | Reverse | TTGTGAAGTAGGGAAGGCCG |
| NLRP3 | Foward | TGTACCCAAGGCTGCTATCT |
|  | Reverse | TTGCAACGGACACTCGTCAT |
| ASC | Foward | GTGGCCCAGTGGTAGAACATA |
|  | Reverse | CAAATGGGGAGCCAGGAATCA |
| MyD-88 | Foward | ACTGATGCGGAGCCAGATTC |
|  | Reverse | TGGGAGGAAAGGCAGTCCTA |
| TLR4 | Foward | GGAAGACAAAAGAAAGACAGCCC |
|  | Reverse | TGGGGAGATTCTTGATCTGCT |
| Collagen-Ⅰ | Foward | GAGCAGACGGGAGTTTCTCCT |
|  | Reverse | CTTCTTGGCCATGCGTCAG |
| Fibronectin-1 | Foward | CGGGAAGGTACTGTCCCATA |
|  | Reverse | GGAAAAGTCCTGAGGTGGGG |

| **Supplementary material Table 2** | | |  |
| --- | --- | --- | --- |
| Primary antibodies for Western Blot and IHC staining. | | |  |
| antibodies | Cat No. | Manufacturer | Sources of species |
| Caspase-1 | ab179515 | abcam | Rabbit |
| Caspase-11 | ab180673 | abcam | Rabbit |
| ASC | ab175449 | abcam | Goat |
| MyD88 | ab2064 | abcam | Rabbit |
| NLRP3 | ab263899 | abcam | Rabbit |
| TLR4 | ab13556 | abcam | Rabbit |
| Fibronectin-1 | ab45688 | abcam | Rabbit |
| Collagen-Ⅰ | ab 270993 | abcam | Rabbit |
| β-Actin | ab115777 | abcam | Rabbit |

| **Supplementary material Table 3** | | |  |
| --- | --- | --- | --- |
| ELISA kits information. |  |  |  |
| Parameters | Cat No. | Manufacturer | species |
| IL-1β | MLB00C | R&D Systems | Mouse |
| IL-6 | M6000B | R&D Systems | Mouse |
| IL-18 | DY7625-05 | R&D Systems | Mouse |

The minimum detectable dose (MDD) of mouse IL-1β ranged from 0.46-4.80 pg/mL.

The minimum detectable dose (MDD) of mouse IL-6 ranged from 1.3-1.8 pg/mL.

The minimum detectable dose (MDD) of mouse IL-18 ranged from 10.5-13.2 pg/mL
