# Supplementary figures and images for: Tetrandrine alleviates silicosis by inhibiting canonical and non-canonical NLRP3 inflammasome activation in lung macrophages
Source: Acta Pharmacol Sin. 2021 Aug 20;43(5):1274–84. doi: 10.1038/s41401-021-00693-6 (PMC9061833; doi:10.1038/s41401-021-00693-6)

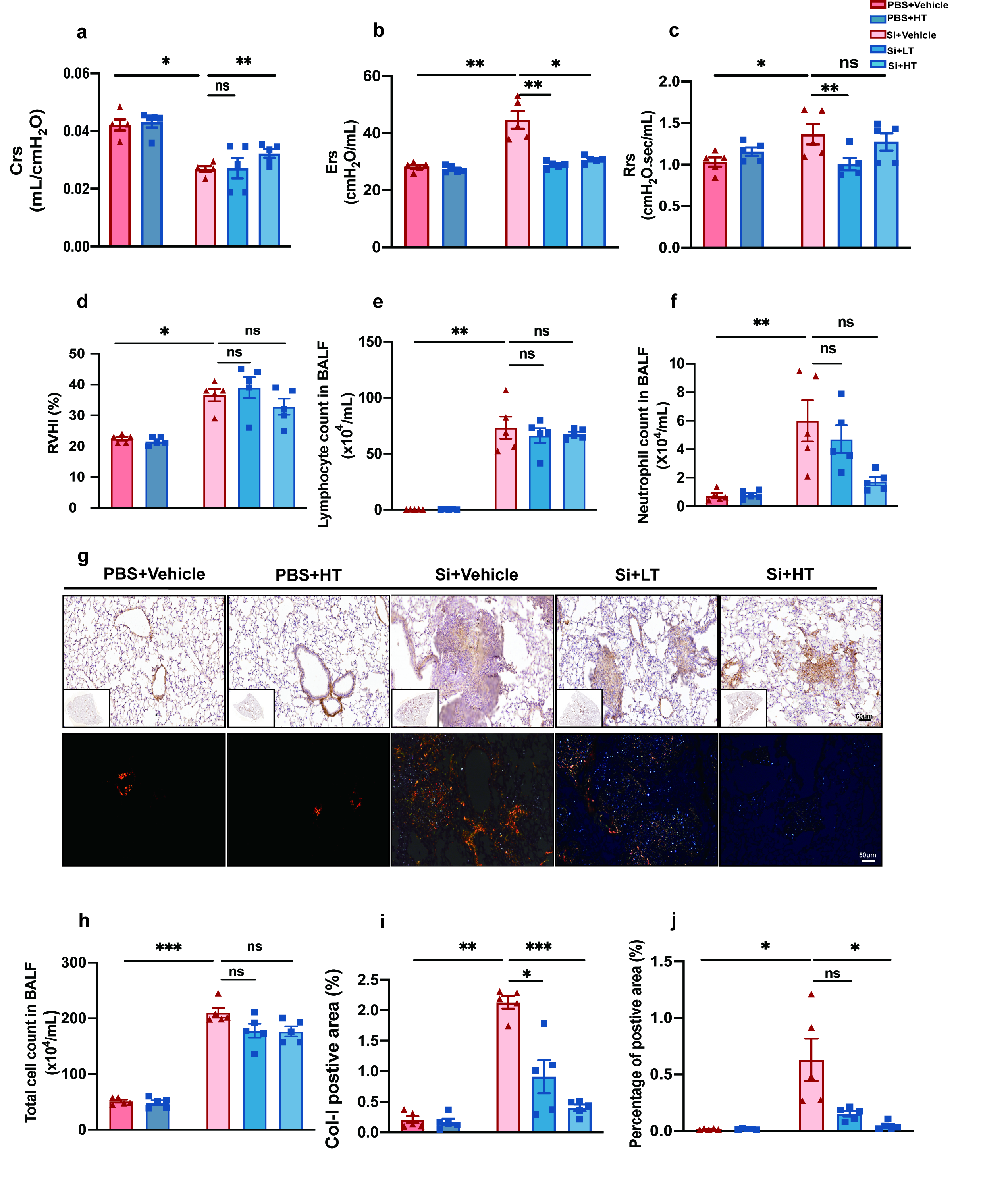

Supplement: Supplementary file 2 — Supplementary Figure S1 [file 41401_2021_693_MOESM2_ESM.tif]

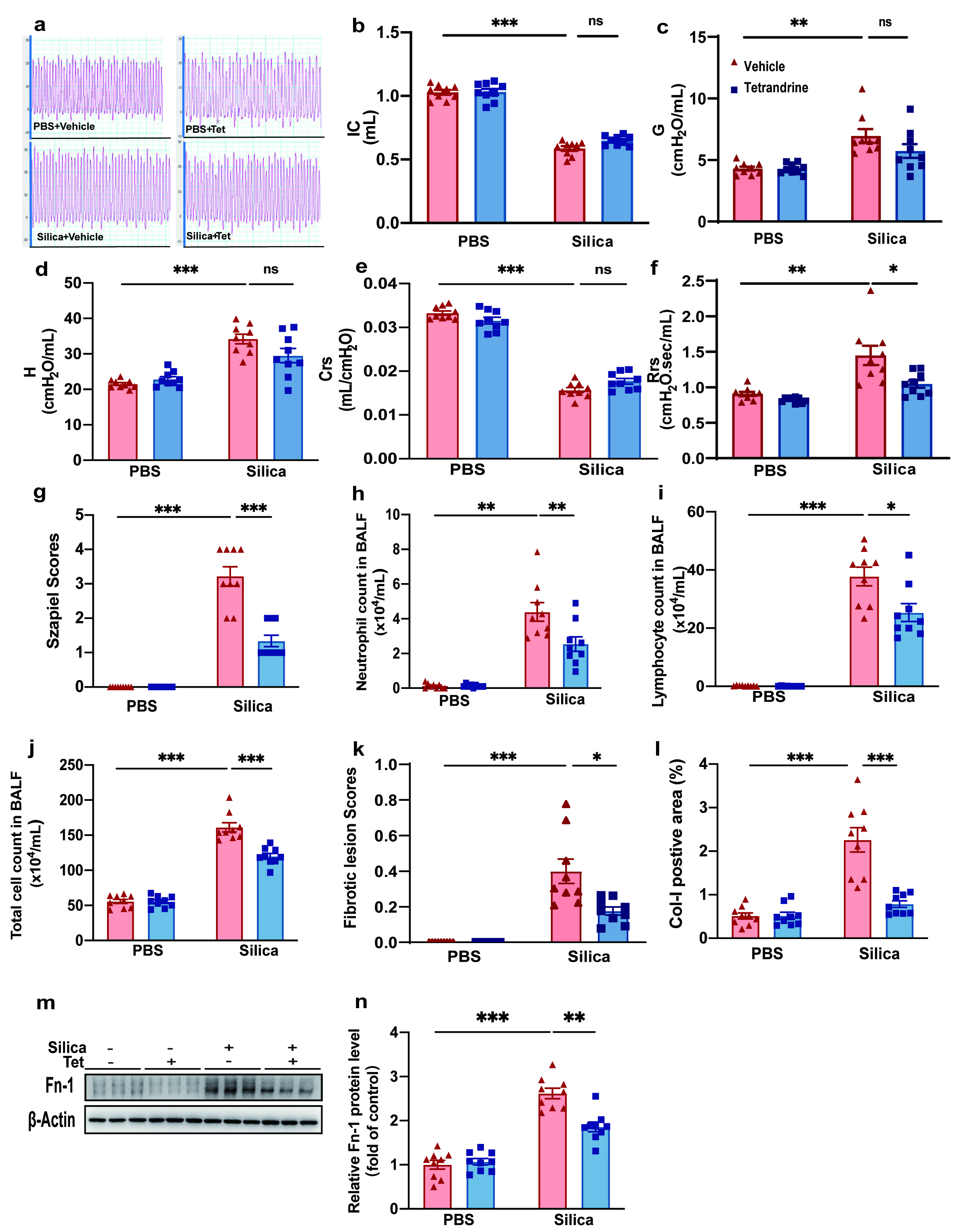

Supplement: Supplementary file 3 — Supplementary Figure S2 [file 41401_2021_693_MOESM3_ESM.tif]
