# Supplementary material for: Tetrandrine alleviates silicosis by inhibiting canonical and non-canonical NLRP3 inflammasome activation in lung macrophages
Source: Acta Pharmacol Sin. 2021 Aug 20;43(5):1274–84. doi: 10.1038/s41401-021-00693-6 (PMC9061833; doi:10.1038/s41401-021-00693-6)
Supplement: Supplementary file 4 — Supplementary Figure legend [file 41401_2021_693_MOESM4_ESM.docx]

**Fig.S1 Tet treatment improved lung impairment, pulmonary inflammation and fibrosis in an early therapeutic silicosis mouse model. (a-c)** Lung function test of mice treated as in Fig.1(a). Crs, Ers and Rrs, compliance, elasticity and resistance of the whole respiratory system, respectively. **(d)** Right ventricular hypertrophy index (RVHI) of mice treated as in Fig.1(a). **(e)** Lymphocyte and **(f)** neutrophil count in BALF of mice treated as in Fig.1(a). **(g)** Immunohistochemical (IHC) staining of Col-I (above) and Sirius red staining (below) of lung sections from mice treated as in Fig.1(a) (n=5 per group). Scale bar, 50 μm. **(h)** Total cell count in BALF from mice treated as in Fig.1(a). **(i)** Statistical analysis of Col-Ⅰ positive area of IHC staining in images of (g). Col-Ⅰ, Collagen-I. **(j)** Statistical analysis of percentage of positive area of Sirius red staining in images of (g). The data are reported as the mean ± SEM. Significance for each figure: * indicates *P* < 0.05, ** indicates *P* < 0.01, *** indicates *P* < 0.001 and ns indicates not significant.

**Fig.S2 Late Tet therapeutic treatment reduced pulmonary inflammation and fibrosis and partially ameliorated impaired lung function in silicosis mice. (a)** The graph of RVSP from mice in four groups (PBS + Vehicle, PBS + Tet, Si + Vehicle, and Si + Tet groups, n=9 per group). **(b-f)** Lung function test of mice, including IC, G, H, Crs, and Rrs. **(g)** Statistical analysis of Szapiel Scores from HE staining in Fig.2(e). **(h)** Neutrophil, **(i)** lymphocyte and **(j)** total cell count in BALF from mice. **(k)** Statistical analysis of Fibrotic lesion Scores from Masson staining in Fig.2(j). **(l)** Statistical analysis of Col-Ⅰ positive area from IHC staining in Fig.2(i). **(m)** Western blot of Fn-1 of lung tissue from mice. **(n)** Statistical analysis of band densities from images in (m). The data are reported as the mean ± SEM. Significance for each figure: * indicates *P* < 0.05, ** indicates *P* < 0.01, *** indicates *P* < 0.001 and ns indicates not significant.
